# Effectiveness of spa therapy for patients with chronic low back pain: An updated systematic review and meta-analysis

**DOI:** 10.1097/MD.0000000000017092

**Published:** 2019-09-13

**Authors:** Ruixue Bai, Chihua Li, Yangxue Xiao, Manoj Sharma, Fan Zhang, Yong Zhao

**Affiliations:** a School of Public Health and Management; b Research Center for Medicine and Social Development; c Innovation Center for Social Risk Governance in Health; d Health Management (Physical Examination) Center, The Second Affiliated Hospital, Chongqing Medical University, Chongqing; e Zhengzhou Central Hospital Affiliated to Zhengzhou University, Henan, China; f Department of Epidemiology, Mailman School of Public Health, Columbia University, New York, NY; g Department of Behavioral and Environmental Health, Jackson State University, Jackson, MS.

**Keywords:** chronic low back pain, meta-analysis, spa therapy, systematic review

## Abstract

**Background:**

Low back pain (LBP) is a major health problem around the world. Two previous meta-analyses showed that the spa therapy has a positive effect on reducing pain among patients with LBP based on studies published before 2006 and studies published between 2006 and 2013. In recent years, more studies reported the effect of spa therapy on treating chronic low back pain (CLBP). Our study aimed to update the meta-analysis of randomized controlled trials (RCTs) about the effect of spa therapy on treating CLBP and to examine the effect of spa therapy based on different interventions.

**Methods:**

PubMed, Embase, Web of Science, and Cochrane Library were searched until May 2018 to identify RCTs about spa therapy among patients with CLBP. Summary effect estimates were calculated by using a random-effects model. The quality of each eligible study was evaluated by Jadad checklist.

**Results:**

Twelve studies met the inclusion criteria for the systematic review and were included in meta-analysis. There was a significant decrease in pain based on visual analogue scale (VAS) (mean difference [MD] 16.07, 95% confidence interval [CI] [9.57, 22.57], *P* < .00001, *I*^*2*^ = 88%, n = 966), and lumbar spine function in Oswestry disability index (ODI) (MD 7.12, 95% CI [3.77, 10.47], *P* < .00001, *I*^*2*^ = 87%, n = 468) comparing spa therapy group to control group. Methodological assessment for included studies showed that the study's quality is associated with lacking blinding.

**Conclusion:**

This updated meta-analysis confirmed that spa therapy can benefit pain reliving and improve lumbar spine function among patients with CLBP. Physiotherapy of subgroup analysis indicated that it can improve lumbar spine function. However, these conclusions should be treated with caution due to limited studies. More high-quality RCTs with double-blind design, larger sample size, and longer follow-up should be employed to improve the validity of study results.

## Abstract 2

Supplemental Digital Content is available in the text

## 1 Introduction

Low back pain (LBP) is a major health problem around the world, with an estimated prevalence of around 7.0%.^[1,2]^ The majority of adults (60%–80%) have medical complaints on LBP at some time point in their lives,^[3,4]^ and 5% to 10% of them will further develop chronic low back pain (CLBP).^[5]^ CLBP patients can have symptoms of LBP for over 3 months,^[6–8]^ and elder people, women, domestic workers, and people with higher body mass index are more likely to have CLBP.^[9–14]^ Patients with CLBP may face heavy burden and suffer from long time incapacity, which is accompanied by repeated treatment and social support.^[2,15–18]^

Different methods can be applied for treatment and management of CLBP, including pharmacological and nonpharmacological treatments.^[19]^ Spa therapy is a nonpharmacological and widely used treatment,^[20]^ in which patients bath in natural spring water with a temperature over 20°C and rich mineral contents for 20 to 30 minutes (min). In a broad sense, spa therapy comprises therapeutic modalities including balneotherapy, mud-pack therapy, massage, and supervised water exercises in spa resorts, adding other benefits such as a pleasant climate, relaxing natural scenery, and clean air.^[21,22]^ It is an ancient way to treat rheumatic and musculoskeletal disorders which can relieve the pain and improve the function in musculoskeletal disorders,^[23–26]^ but the mechanism has not been clearly illuminated.^[27,28]^ It may associate with hydrostatic pressure, mineral composition, and temperature.^[23,29,30]^ Immersing in warm water may contribute to an analgesic effect by thermal effect and hydrostatic pressure of water on the skin according to the “Gate control theory of pain.”^[31]^ And due to a lower specific heat, mud-pack therapy elevates the body-core temperature more efficiently.^[32]^ In addition, exercise or physical activity is vital for CLBP patients to help them complete their daily activities by enhancing muscle strength, increasing aerobic capacity of lumbar muscles, and promoting local blood flow.^[23,24,33,34]^ Using spa therapy for managing CLBP is a Grade B recommendation.^[35]^

In 2006, Pittler et al^[20]^ performed a meta-analysis about the effect of spa therapy among patients with LBP, and concluded that spa therapy has a positive effect in pain relieving based on 5 studies. A later systematic review summarized the studies published between 2005 and 2013 and reported the positive effects of spa therapy in treating CLBP.^[36]^ Considering different additional intervention methods may affect therapeutic effects; therefore, we conducted a systematic review and meta-analysis to provide an updated overview of the literature in this area and to further assess short-term effect of spa therapy in patients with CLBP with a more detailed classification on intervention methods of 3 subgroups: balneotherapy, balneotherapy with mud pack, and balneotherapy with physiotherapy.

## 2 Materials and methods

This study was performed according to the statement, preferred reporting items for systematic reviews and meta-analyses (PRISMA)^[37]^ and recommendations of the Cochrane Collaboration.^[38]^ All analyses were conducted based on previously published studies, so no ethical approval and patient consent are required.

### 2.1 Search strategy

The study used the following words as search terms: “spa therapy,” “balneotherapy,” “balneology,” “hot spring,” and “geothermal spring” combine with “low back pain” and “lumbago” in PubMed, Embase, Web of Science, and Cochrane Library. Each database was searched from its inception to May 2018. Two authors (R.B. and C.L.) screened independently. The search strategy applied a combination of title and abstract, and used the Mesh Term. Hand searching is performed by reviewing the references of included studies.

### 2.2 Study selection

Titles and abstracts of identified articles were reviewed by 2 authors (R.B. and C.L.) independently. When 2 reviewers could not reach a consensus, disagreements and uncertainties were resolved through discussion. The including criteria were: patients who were diagnosed with CLBP, treated with spa therapy in a randomized way [randomized clinical trials (RCTs)], clinical trials whose main objectives included the effectiveness of spa therapy, intervention for spa therapy applied as a combination of balneotherapy with physiotherapy, mud-pack, publications in English only. Exclusion criteria were: the mineral water was not natural spring, spa therapy intervention lasted for more than 3 months.

### 2.3 Data extraction

Two reviewers (R.B. and C.L.) extracted the following data from all included studies independently: article information: authors’ names, and publication time, reported study characteristics: course of the treatment, overall follow-up duration, characteristics of the thermal water: geographical area, composition, mineral concentration, and temperature, intervention and control group: method of therapy, duration, and frequency, observing parameters: visual analogue scale (VAS), Schober test, and Oswestry disability index (ODI), outcome measurements: the evaluation of outcome.

### 2.4 Methodological quality assessment

Jadad checklist was used to evaluate included studies on different aspects, including treatment methods relevant to the description of randomization, double-blind structure, and withdrawals/dropouts.^[39]^ The range of quality score is from 0 to 5 (the lowest to highest). Studies with a score of or over 3 were regarded as having a good quality. Two reviewers (R.B. and C.L.) assessed the quality of included studies independently. Disagreements were resolved through discussion until reaching a consensus.

### 2.5 Statistics analysis

VAS, Schober test, and ODI evaluate the intensity of pain, lumbar spine mobility, and lumbar spine function respectively, and they were chosen as main outcome measures for meta-analysis. In some included studies, these measures were examined for several times at different time points. The data at the first time point after treatment and/or in the rest condition were used for analysis. All the quantitative data were converted into millimeter unit. The random effects model was applied to generate summary estimates. Heterogeneity was assessed by *I*^2^ test. When *I*^2^ < 25%, it means no heterogeneity; when 25% ≤*I*^2^ < 50%, it means moderate heterogeneity. The heterogeneity is acceptable; when *I*^2^≥50%, it means strong heterogeneity. Subgroup and sensitivity analysis were used to examine the source of heterogeneity.^[40]^ Funnel plots were used to assess publication bias. All statistical analyses were conducted in Review Manager (version 5.2).

## 3 Results

### 3.1 Study selection

**Figure 1:**
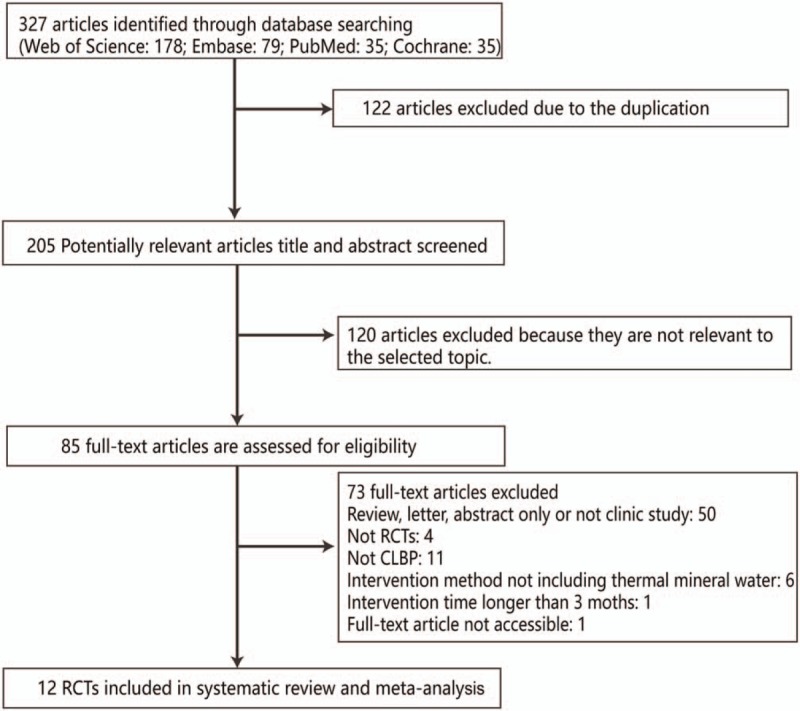
Study selecting flowchart.

A total of 327 studies were initially retrieved from databases, and 12 RCT studies met the eligibility criteria and were included, and their data were assessed in the meta-analysis (Fig. 1).

### 3.2 Study characteristics

The characteristics of included studies were summarized in Table 1   . Based on their intervention methods, 3 were balneotherapy,^[41–43]^ 2 were balneotherapy with mud-pack therapy,^[44,45]^ and 7 were balneotherapy with physiotherapy.^[46–52]^ The length of treatment in most trials was around 3 weeks.^[41,42,44–46,48–51]^ The follow-up efficacy of spa-therapy was observed in 8 trials.^[41–45,47,50–52]^ Most of them reported a significant improvement in pain relief, lumbar flexibility, functional capacity, and quality of life. No adverse events were reported in all included trials. These studies were performed in Hungary,^[42,43,50,51]^ Turkey,^[46–48,52]^ France,^[41,44,45]^ and Croatia^[49]^ and the temperature of spa therapy was between 31°C and 38°C.

**Table 1:**
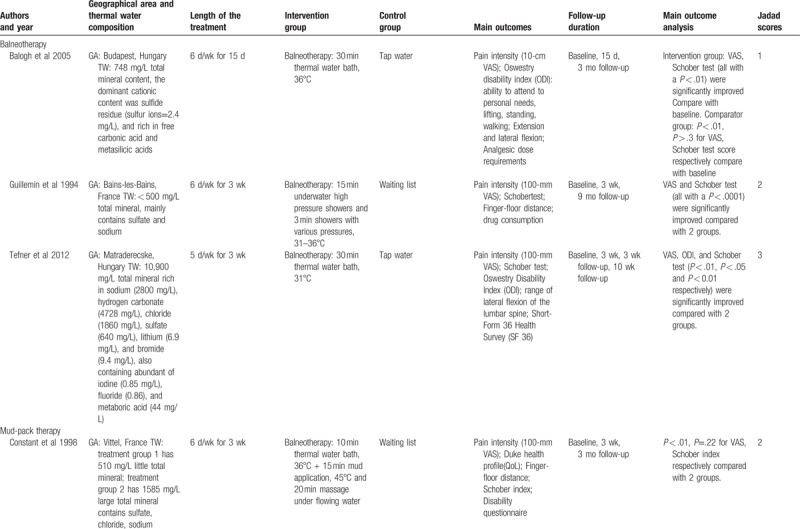
Characteristics of the included studies.

**Table 1 (Continued):**
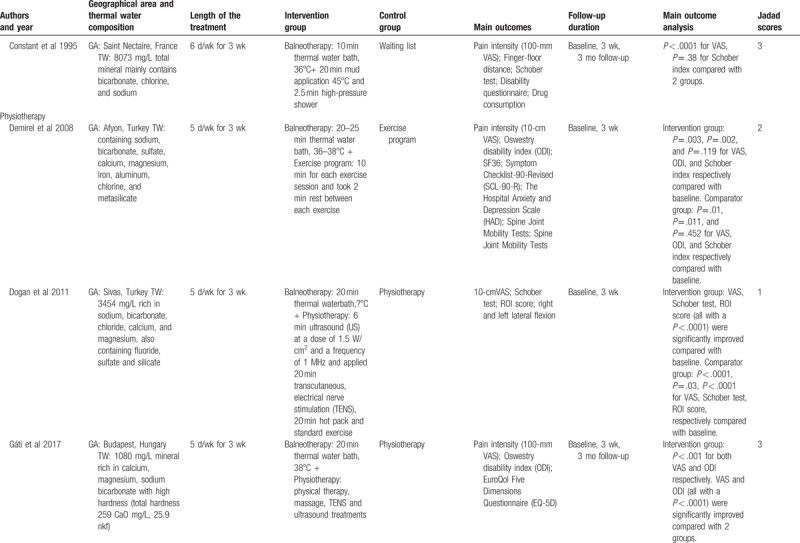
Characteristics of the included studies.

**Table 1 (Continued):**
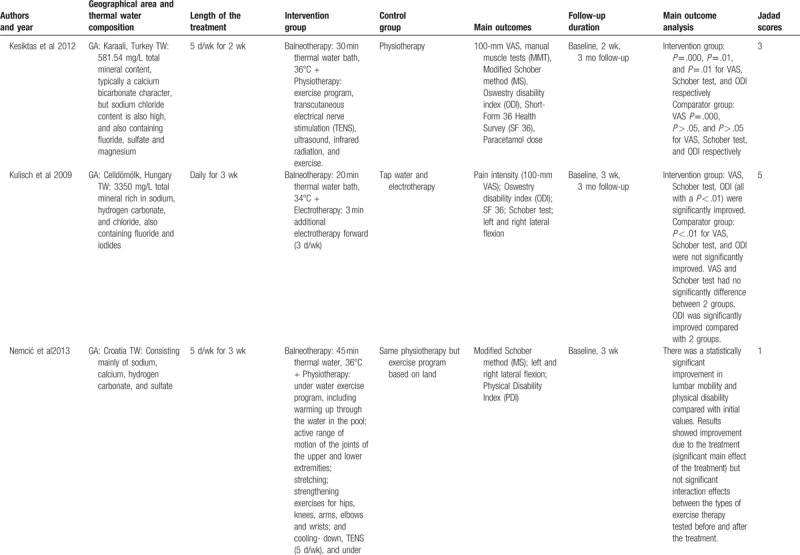
Characteristics of the included studies.

**Table 1 (Continued):**
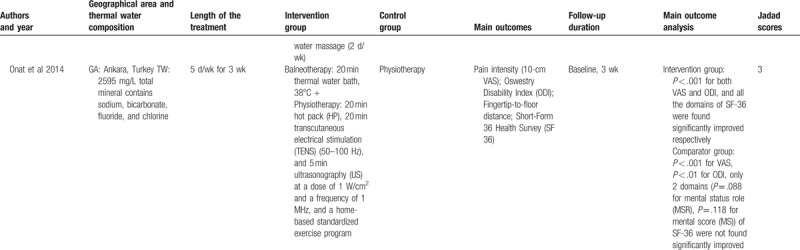
Characteristics of the included studies.

### 3.3 Meta-analysis

**Figure 2:**
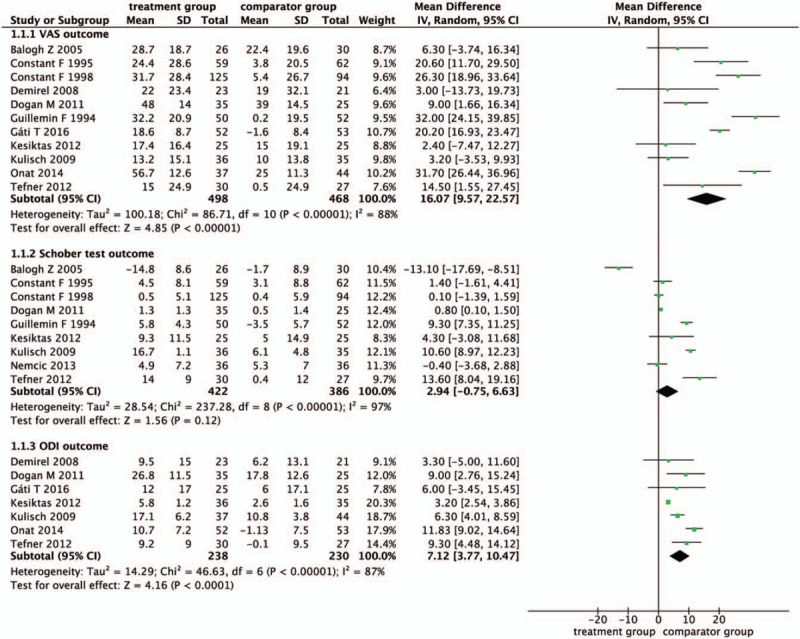
Effect estimates on included studies comparing thermal water with control.

Overall, 11 studies were included in meta-analysis.^[41–48,50–52]^ In Figure 2, 966, 808, and 468 patients with data on VAS, Schober tests, and ODI respectively were included in data synthesis. In respect of effectiveness of spa therapy for CLBP, there was statistical significance between treatment and control group in VAS (mean difference [MD] 16.07, 95% confidence interval [CI] [9.57, 22.57], *P* < .00001, *I*^*2*^ = 88%, n = 966), and ODI (MD 7.12, 95% CI [3.77, 10.47], *P* < .00001, *I*^*2*^ = 87%, n = 468). No statistically significance was found in Schober test (MD 2.94, 95% CI [−0.75, 6.63], *P* < .00001, *I*^*2*^ = 97%, n = 808).

### 3.4 Subgroup analyses

According to treatment method, 3 subgroups were divided: balneotherapy group, balneotherapy with mud-pack therapy group, and balneotherapy with physiotherapy group. No ODI data were collected in balneotherapy group, results shown in Supplementary Figure 1, heterogeneity of VAS, and Schober test were still huge. No ODI data were collected in balneotherapy with mud-pack therapy group. Only VAS had statistical significance (MD 23.99, 95% CI [18.33, 29.66], *P* < .00001, *I*^*2*^ = 0%, n = 340), shown in Supplementary Figure 2. Supplementary Figure 3 showed results of balneotherapy with physiotherapy group. However, heterogeneities in VAS, Schober test, and ODI were significant.

### 3.5 Sensitivity analyses

Lower heterogeneity was observed in results of spa therapy ODI (*I*^*2*^ = 87–54%) after excluding the study of Kulisch.^[51]^ After excluding 1 study from the balneotherapy and physiotherapy of subgroup, the heterogeneity decreased dramatically in all outcomes indicators, shown in Supplementary Figure 4.

### 3.6 Quality assessment

For quality evaluation, 6 studies have good quality^[42,44,47,48,50,51]^ with only 1 trial having a full score.^[51]^ Other trials showed low quality: 3 scored 1 point,^[43,46,49]^ and 3 had 2 points.^[41,45,52]^

## 4 Discussion

Our meta-analysis examined the effect of short-term spa therapy on pain relief and lumbar spine function improvement among patients with CLBP. Although spa therapy has been widely used in the world, especially in Europe, comprehensive and concrete evidence is still needed to verify its effectiveness for CLBP. Compared to previous meta-analysis and review publications,^[20,36]^ the present meta-analysis included more studies, examined more outcome measurements including lumbar spine mobility (Schober test) and lumbar spine function (ODI), and stratified analysis based on different intervention methods: balneotherapy,^[41–43]^ balneotherapy with mud-pack,^[44,45]^ and balneotherapy with physiotherapy.^[46–52]^

In subgroup and sensitivity analysis, VAS's improvement in treatment group was significantly higher than control group, which is consistent with the findings reported by Pittler et al.^[20]^ Significant heterogeneity was observed in balneotherapy and balneotherapy with physiotherapy group. In sensitivity analyses, heterogeneity decreased, Schober tests variations increased in both subgroups. In balneotherapy group, heterogeneity may be explained by differences in study design between Guillemin et al^[41]^ and others.^[42,43]^ In balneotherapy with physiotherapy group, Gáti et al's and Kulisch et al's studies^[50,51]^ were conducted in Hungary, and other studies^[46–49,52]^ were conducted in Turkey and Croatia, all of latter were Mediterranean countries. In addition, Kulisch's study^[51]^ had the full scores of Jadad check list, the heterogeneity across included studies decreased after its exclusion, which may be due to the overall methodology inconsistencies. Specifically, this trial used tap water combined with physiotherapy was performed as control group, different from other physiotherapy studies. However, in balneotherapy with mud-pack group, there was no significant difference in Schober test variation between treatment and control group. The treatment durations of both studies in this group were relatively short, which were around 10 minutes thermal mineral water bath. Generally, mean duration of balneotherapy was 20 minutes to 30 minutes. Thus, this experimental design may lead to an incomplete demonstration in the effectiveness of spa therapy. Otherwise, although ODIs were significantly decreased in subgroup analysis and sensitivity analysis, significant heterogeneities could not be neglected. Indeed, ODI is a patient self-rated scale with greater subjectivity, while Schober test is more objective. In addition to pain alleviating in patients with CLBP, spa therapy also improves lumbar mobility.

We evaluated the short-term spa therapy effect. Eight trials evaluated the follow-up efficacy,^[41–43,45,47,50,51]^ and most of them lasted for 3 months, except one was 6 months^[44]^ and the other was 9 months.^[41]^ After follow-up, most studies have observed that VAS significantly decreased compared with the baseline levels, and the Schober index and ODI also improved significantly. There were significant differences in the drop of VAS scores between the intervention and control group. In Guillemin et al,^[41]^ the authors used spa therapy as an intervention group, while the control group only allowed painkillers. After 9 months of follow-up, the authors observed that results based on VAS and Schober test significantly improved. But, considering the control group did not receive any treatment in 9 months, symptoms might become more severe. The authors believed that this may lead to an overestimation in the long-term therapeutic effect of spa therapy. While the short-term effect of spa therapy is well known, its long-term benefit is still under discussion because of the paucity evidence. Compared to the baseline, 7 trials observed that the VAS of spa therapy group was significantly decreased.^[43,46–48,50–52]^ The effect of spa therapy on Schober index and ODI is controversial: most researchers suggested that the spa therapy could ameliorate the lumber function or mobility after the treatment,^[43,46–48,50,51]^ although other researchers did not find the improvement.^[52]^ Meanwhile, some trials used therapeutic methods in control groups because of ethical reasons, such as hydrotherapy,^[42,43,51]^ physiotherapy.^[46,47,50,51]^ In these studies, VAS was also significantly lower than the baseline. Hydrotherapy, exercise therapy, as well as the physiotherapy, also has therapeutic effects.^[53]^ These designs will influence results of the studies. Although Tefner et al^[42]^ observed that the VAS and range of motion significantly improved and differed between groups, there was no statistical difference in Kulisch et al's study.^[51]^

No adverse events were reported in included studies and adverse events in spa therapy are rarely reported. Previous studies pointed out that the most common adverse event was respiratory tract infections (8%), which were more common among patients with chronic respiratory failure and chronic bronchitis.^[35,54]^ Other common adverse events include mild neurological disorders (6%), pain exacerbation (5%), skin diseases (2%), falls (1%), urinary tract infections (<1%), cardiovascular disorders, and erysipelas (0.005%) and should also be paid attention to.

Up to now, there is no guideline about spa therapy. According to designs of included studies, we recommend that the duration of spa therapy should longer than 30 minutes; temperature should be higher than 38°C. Besides, patients with following conditions are not suggested to receive spa therapy: acute infection, pregnancy, cardiovascular diseases (such as heart failure, unstable hypertension, angina pectoris), respiratory insufficiency, uncontrolled liver disorders, uncontrolled and unstable metabolic disorders, epilepsy, and uncontrolled epilepsy.^[17,45,52]^

Interestingly, all included studies in our review were conducted in Europe (Hungary, Turkey, France, and Croatia). The first trial about spa therapy who used double blind and tap water control was performed in Hungary, was applied among patients with rheumatoid arthritis. It might be because that in other countries, people go to spas not only for health but also for recreation and rest.^[55]^

As for the methodological assessment, there was only 1 full marks study.^[51]^ Interestingly, in subgroup analysis and sensitivity analysis, after exclusions of this study, we found the heterogeneity declined, maybe the inconsistencies of study methods cause the heterogeneity, especially the missing designs of double-blind study design. However, it is difficult to execute blinding because of special smell of spa water. Therefore, RCTs with more rigorous double-blind design are needed.

There are certain limitations in this meta-analysis. First, all included studies were only published in English, whereas in this filed the majority of the studies were conducted in Europe, so studies published in other languages cannot be analyzed in this meta-analysis. This may contribute to publication bias. Second, heterogeneity in results was considerable. We ascribed this to poor designs and excessive time gap of included studies. Most studies reported unclear randomization and insufficient double-blind design. Further research with high-quality RCTs was required. Furthermore, the sample size in all the included studies was small (<100 per treatment arm). The small number of studies and participants included would result in an underpowered analysis. These included studies’ published time spanned over 24 years, and the excessive time gap that might reduce the homogeneity of participants. In addition, more parameters are needed to evaluate and verify the efficacy of spa therapy, and the long-term efficacy should be confirmed.

## 5 Conclusion

In conclusion, this updated systematic review and meta-analysis demonstrated that spa therapy may have short-term beneficial effects on pain reliving and lumbar spine mobility improvement in patients with CLBP. This meta-analysis provides recommendations for future research: more rigorous study design, longer follow-up period, and bigger sample size to provide more convinced evidence in spa therapy to treat CLBP.

## Acknowledgments

Thanks for Hongtao Tie and Guochao Zhong of Chongqing Medical University who modified the manuscript and gave us some advice, and Xinran Lai of Monash University who modified language and grammar for this study.

## Author contributions

Conceptualization: Ruixue Bai, Chihua Li, Fan Zhang, Yong Zhao.

Methodology: Ruixue Bai, Chihua Li, Fan Zhang, Yong Zhao.

Data curation: Ruixue Bai, Chihua Li, Yong Zhao.

Validation: Ruixue Bai, Chihua Li, Yangxue Xiao, Fan Zhang.

Writing – review & editing: Ruixue Bai, Chihua Li, Yang-xue Xiao, Manoj Sharma, Fan Zhang, Yong Zhao.

**Conceptualization:** Manoj Sharma, Fan Zhang, Yong Zhao.

**Data curation:** Ruixue Bai.

**Formal analysis:** Ruixue Bai, Chihua Li.

**Funding acquisition:** Yong Zhao.

**Methodology:** Fan Zhang, Yong Zhao.

**Project administration:** Fan Zhang, Yong Zhao.

**Software:** Ruixue Bai.

**Supervision:** Fan Zhang, Yong Zhao.

**Writing – original draft:** Ruixue Bai, Chihua Li.

**Writing – review & editing:** Ruixue Bai, Chihua Li, Yangxue Xiao, Manoj Sharma, Fan Zhang, Yong Zhao.

Ruixue Bai orcid: 0000-0001-6043-3877.

## Supplementary Material

Supplemental Digital Content
