# Supplementary material for: Effectiveness of spa therapy for patients with chronic low back pain: An updated systematic review and meta-analysis
Source: Medicine (Baltimore). 2019 Sep 13;98(37):e17092. doi: 10.1097/MD.0000000000017092 (PMC6750337; doi:10.1097/MD.0000000000017092)

**Suppl. Figure 1.** Results of balneotherapy subgroup analysis

**
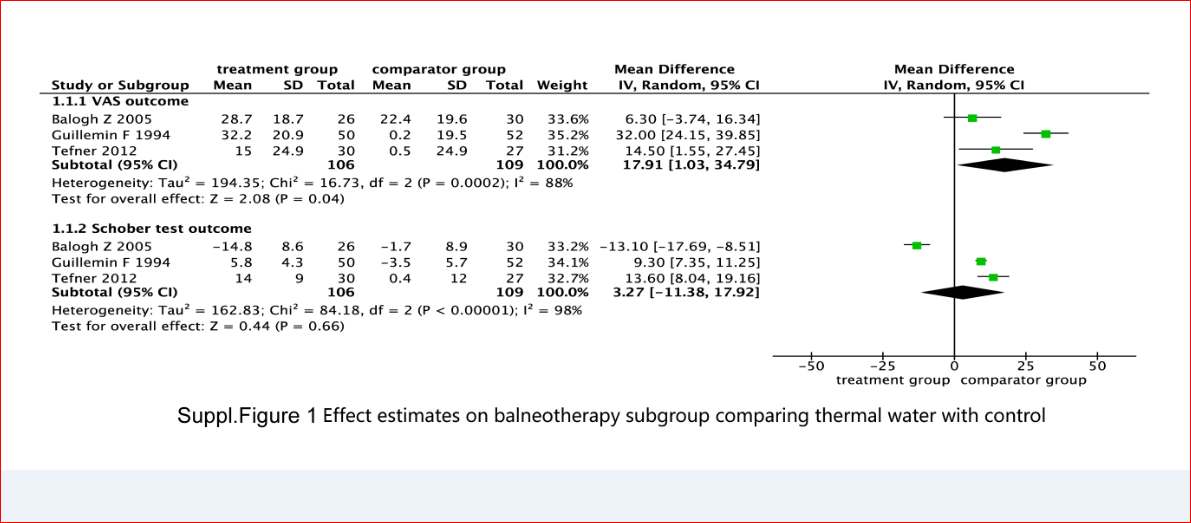
**

**Suppl. Figure 2.** Results of mud-pack subgroup analysis

**
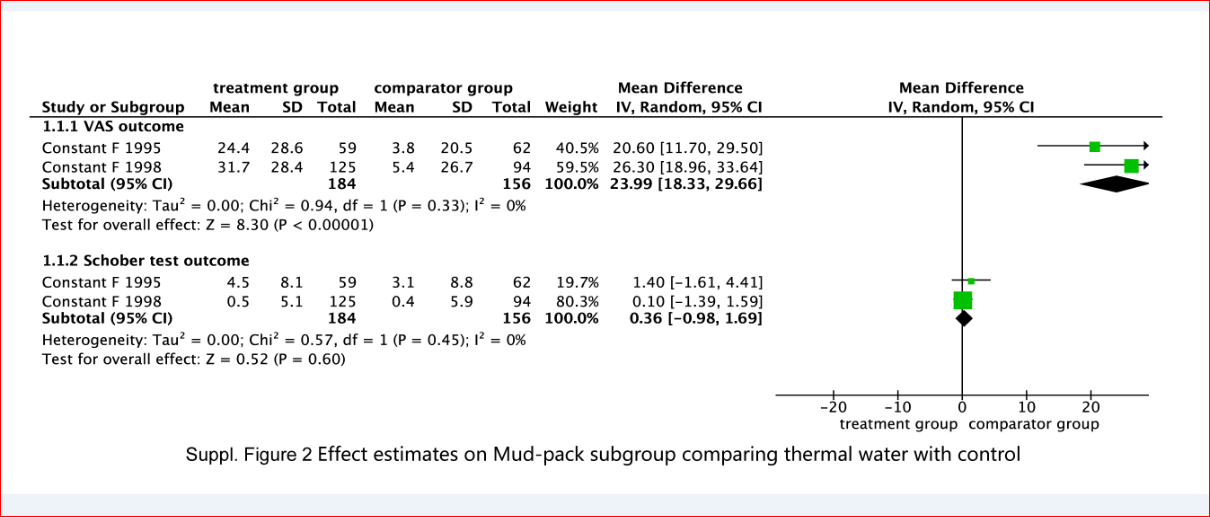
**

**Suppl. Figure 3.** Results of physiotherapy subgroup analysis

**
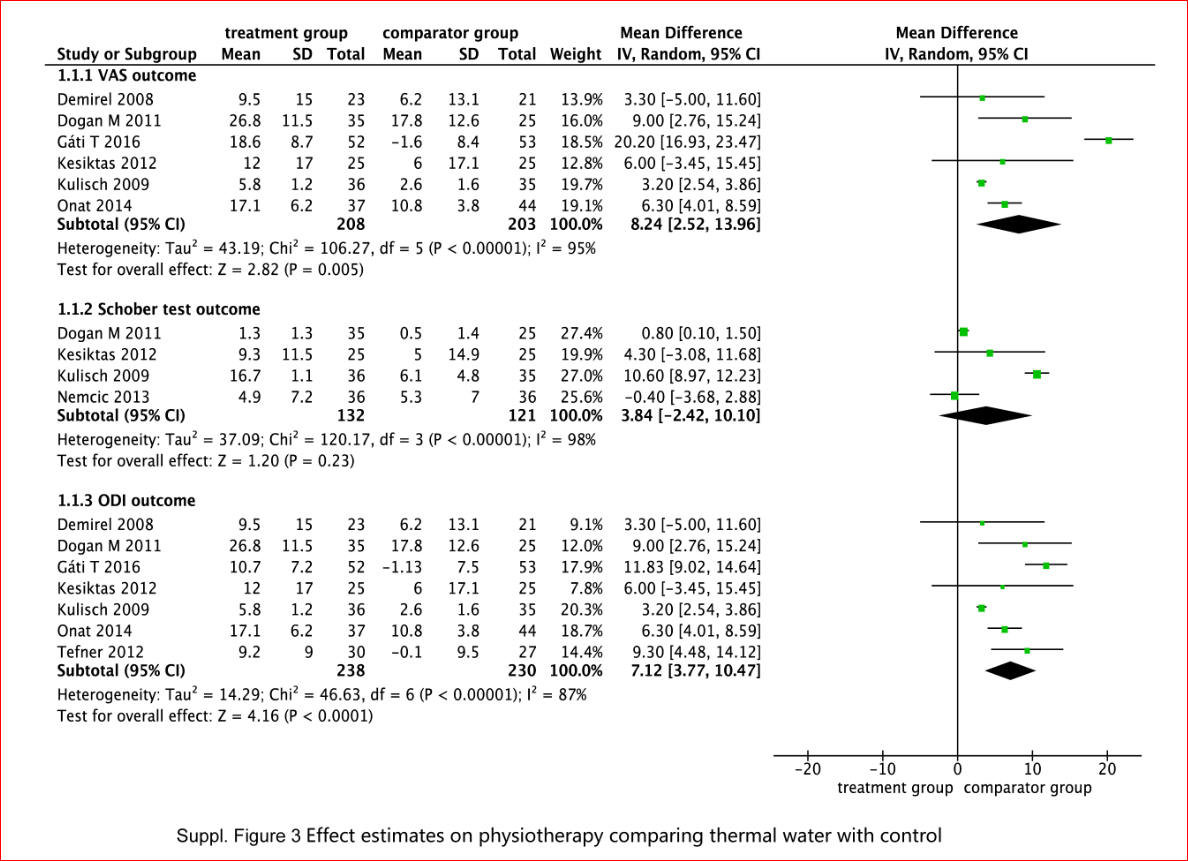
**

**Suppl. Figure 4.** Results of sensitivity analysis


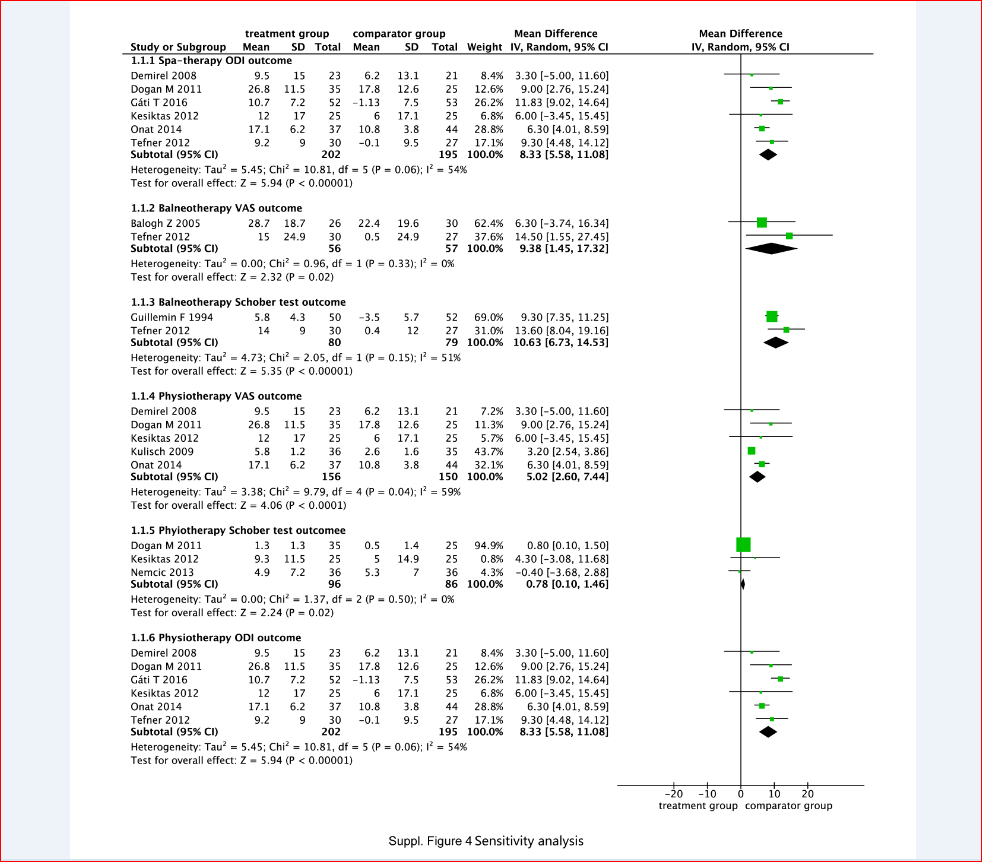

Supplement: Supplemental Digital Content [file medi-98-e17092-s001.doc]
